# Supplementary material for: LSTrAP-Cloud: A User-Friendly Cloud Computing Pipeline to Infer Coexpression Networks
Source: Genes (Basel). 2020 Apr 16;11(4):428. doi: 10.3390/genes11040428 (PMC7230309; doi:10.3390/genes11040428)
Supplement: Supplementary file 1 [file genes-11-00428-s001.zip › Supp_figure1.pdf]

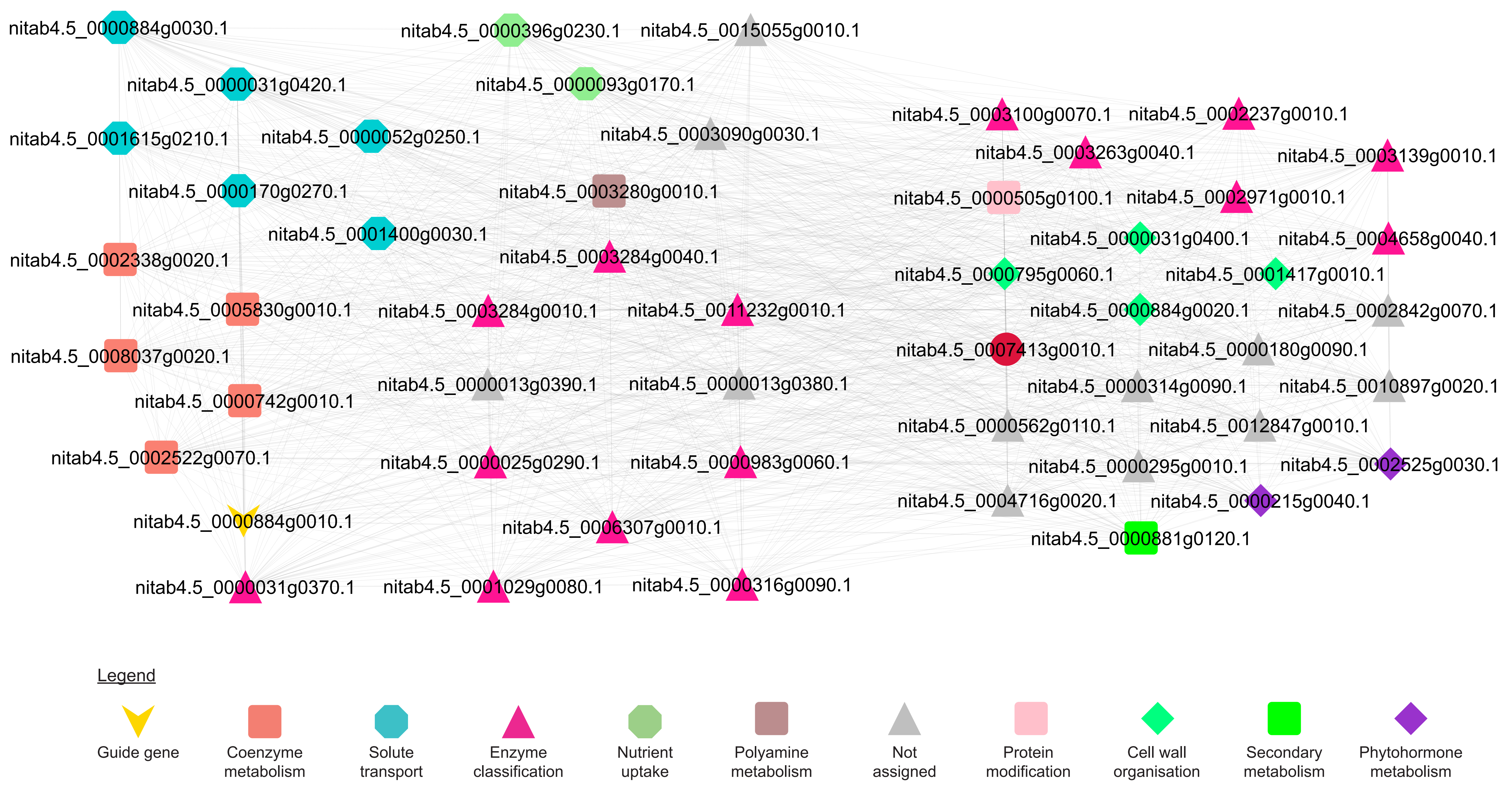
nitab4.5_0000884g0030.1
nitab4.5_0000396g0230.1
nitab4.5_0015055g0010.1
nitab4.5_0000031g0420.1
nitab4.5_0000093g0170.1
nitab4.5_0003100g0070.1
nitab4.5_0002237g0010.1
nitab4.5_0001615g0210.1
nitab4.5_0000052g0250.1
nitab4.5_0003090g0030.1
nitab4.5_0003263g0040.1
nitab4.5_0003139g0010.1
nitab4.5_0000170g0270.1
nitab4.5_0003280g0010.1
nitab4.5_0000505g0100.1
nitab4.5_0002971g0010.1
nitab4.5_0001400g0030.1
nitab4.5_0000031g0400.1
nitab4.5_0004658g0040.1
nitab4.5_0002338g0020.1
nitab4.5_0003284g0040.1
nitab4.5_0000795g0060.1
nitab4.5_0001417g0010.1
nitab4.5_0005830g0010.1
nitab4.5_0003284g0010.1
nitab4.5_0011232g0010.1
nitab4.5_0000884g0020.1
nitab4.5_0002842g0070.1
nitab4.5_0008037g0020.1
nitab4.5_0007413g0010.1
nitab4.5_0000180g0090.1
nitab4.5_0000013g0390.1
nitab4.5_0000013g0380.1
nitab4.5_0000314g0090.1
nitab4.5_0010897g0020.1
nitab4.5_0000742g0010.1
nitab4.5_0000562g0110.1
nitab4.5_0012847g0010.1
nitab4.5_0002522g0070.1
nitab4.5_0000025g0290.1
nitab4.5_0000983g0060.1
nitab4.5_0000295g0010.1
nitab4.5_0002525g0030.1
nitab4.5_0004716g0020.1
nitab4.5_0000215g0040.1
nitab4.5_0000884g0010.1
nitab4.5_0006307g0010.1
nitab4.5_0000881g0120.1
nitab4.5_0000031g0370.1
nitab4.5_0001029g0080.1
nitab4.5_0000316g0090.1
Legend
Guide gene
Coenzyme metabolism
Solute transport
Enzyme classification
Nutrient uptake
Polyamine metabolism
Not assigned
Protein modification
Cell wall organisation
Secondary metabolism
Phytohormone metabolism
